# Waiting to inhale: factors associated with healthcare workers’ fears of occupationally-acquired tuberculosis (TB)

**DOI:** 10.1186/s12879-019-4115-z

**Published:** 2019-05-28

**Authors:** Michelle Engelbrecht, Asta Rau, Gladys Kigozi, André Janse van Rensburg, Edwin Wouters, Nina Sommerland, Caroline Masquillier, Kerry Uebel

**Affiliations:** 1 Centre for Health Systems Research & Development, University of the Free State, Nelson Mandela Road, Bloemfontein, 9300 South Africa; 2 Research Centre for Longitudinal & Life Course Studies (CELLO), University of Antwerp, Sint-Jacobstraat 2, BE-2000 Antwerp, Belgium

**Keywords:** Tuberculosis, Occupationally-acquired, Fear of contagion, Stigma, Healthcare workers

## Abstract

**Background:**

Fear of TB infection is rooted in historical and social memories of the disease, marked by stigma, segregation and exclusion. Healthcare workers (HCWs) face these same fears today, and even seek to hide their TB status when infected. This study sought to investigate factors associated with HCWs fears of acquiring TB while at work, including selected biographic characteristics, TB knowledge, infection control and perceptions that their colleagues stigmatise co-workers with TB/ presumed to have TB.

**Methods:**

In the Free State Province, South Africa, a representative sample of 882 HCWs from eight hospitals completed self-administered questionnaires on issues related to fear of occupationally acquired TB, infection control, TB knowledge and workplace TB stigma. The data were analysed using descriptive statistics as well as binomial logistic regression.

**Results:**

Most of the HCWs (67.2%) were concerned about contracting TB at work. Support staff were less likely to worry about acquiring TB than clinical staff (OR = 0.657, *P* = 0.041). Respondents who indicated that there were inadequate numbers of disposable respirators at work, were 1.6 times more likely to be afraid of contracting TB at work (*P* = 0.040). With every unit increase on the TB stigma scale, respondents were 1.1 times more likely to fear acquiring TB at work (*P* = 0.000).

**Conclusions:**

Being a professional clinical HCW, not having adequate disposable respirators available and seeing/perceiving co-workers stigmatise colleagues with (presumptive) TB were all significantly associated with the fear of occupationally-acquired TB. It is recommended that campaigns to destigmatise TB, as well as appropriate TB infection control education and measures, are necessary to alleviate HCWs fears of acquiring the disease in the workplace. Ultimately this should create a health-enabling working environment, where HCWs are not afraid to function and are free to seek treatment and support when necessary.

## Background

Healthcare workers (HCWs) are at the forefront of the battle against tuberculosis (TB), a disease that – because it is airborne – creates a precarious working environment for them. This is especially the case in low- and middle-income countries with a high TB prevalence, where HCWs are at an increased risk of infection due to being exposed to greater numbers of TB patients [1] over long periods of time [2, 3]. Poorly implemented, and sometimes even absent, infection control measures [1, 4–12] as well as a high prevalence of undiagnosed TB in healthcare facilities, further compound the risk to HCWs of TB infection [1].

There is very strong evidence that, for HCWs, TB is an occupationally-acquired disease. Firstly, there is a high prevalence of *latent* TB infection (LTBI) among HCWs [13] compared to the general population. According to World Health Organization (WHO) [13] estimates, the notification rate for *active* TB among HCWs in South Africa in 2015 was 1565 per 100,000 — more than double the notification rate in the general adult population. Earlier research found that HCWs may even be up to three times more likely to acquire TB than the general population [14]. Furthermore, they are six times more likely to be hospitalised for drug-resistant TB (DRTB) than the population they care for [15]. As a meta-analysis reveals: 81% of TB cases among HCWs in high TB incidence countries were attributable to exposure in healthcare settings [2], with all cadres of HCWs being effected including clinical, paramedical/allied, support and administrative staff [16].

Research has found that HCWs are indeed afraid of contracting TB [7, 17], particularly DRTB [18, 19]. Some reasons for their fears include: childcare responsibilities at home and infecting other family members, prolonged treatment and side effects, and workplace and social stigma associated with TB [17, 18]. Von Delft et al. [20] note that while many HCWs are rightfully afraid of contracting TB in the workplace, they develop psychological defence and coping mechanisms. They illustrate this with the example of “battle-hardened” seniors who pass on their feelings of invincibility to younger cadres of staff. The increasing number of HCWs diagnosed with TB, particularly DRTB, leads to tension between denial and the recognition of danger in the workplace.

Against the backdrop of an unremitting global shortage of HCWs [21], every attempt should be made to protect and keep this scarce resource safe. In high TB burden settings, HCWs become less motivated to work in high risk areas, and in some instances even consider leaving the healthcare profession [18]. This renders knowledge on the drivers of this fear highly relevant as certain individual and organizational characteristics – independent from the actual risk of infection due to poor infection control measures – make HCWs more vulnerable to this fear of TB infection [22]. This paper therefore seeks to describe factors associated with HCWs’ fear of contracting TB in public hospitals in the Free State Province. More specifically, we investigated the association between selected biographical variables, TB knowledge, infection control, perceptions that colleagues stigmatise co-workers with TB/presumed to have TB and the fear of occupationally acquired TB.

## Methods

### Design

The paper utilises pre-intervention data from a cluster randomised controlled trial entitled *Towards a health-enabling working environment: developing and testing interventions to decrease HIV- and TB-stigma among healthcare workers in the Free State, South Africa* [23]*.* The trial’s unique focus is on HIV- and TB-stigma *by* HCWs *towards* HCWs — it investigates stigma and associated issues within the workforce, not between HCWs and patients. HCWs are defined as *all* people, working in *all* departments, and in *all* jobs or professions in a hospital.

### Setting and sample

The study setting was public hospitals in the Free State Province, South Africa. From the total population of 28 hospitals that could be selected as comparators, one was eliminated because it was the site of an earlier pilot study [24]. To ensure an equal distribution of large, medium and small hospitals across the Province, hospitals of similar size (based on numbers of staff), were grouped together. The number of hospitals required was estimated to be ±8: two large hospitals (*n* = 6), two medium hospitals (*n* = 8) and four small hospitals (*n* = 13). The hospitals were paired according to size and within these pairs, a coin toss was used to randomly allocate hospitals to Arm A or Arm B, and then again to allocate the 2 arms to intervention or control. The sample size calculations for hospitals and the number of respondents were estimated based on an earlier pilot study, published elsewhere [24].

A sampling frame for individual respondents (and possible replacements) was drawn up for each of the eight randomly sampled hospitals. Based on our previous experience of fieldwork in Free State hospitals [8], we estimated that the baseline survey would require 50% oversampling of respondents to allow for loss-to-follow-up between the pre- and post-intervention surveys. The resulting sample of 882 HCWs were randomly selected proportionate to the overall size of three occupational categories – as defined in the earlier pilot study [24] – in each hospital. Firstly, clinical professionals (including doctors, all categories of nurses and allied healthcare workers such as dieticians, physiotherapists, pharmacists and social workers) made up 63.9% of respondents (*n* = 446). Secondly, management and administrative staff constituted 13.2% of respondents (*n* = 116). Thirdly, support staff (including porters, messengers, as well as staff from housekeeping, catering and workshop) comprised 36.1% of respondents (*n* = 318). Two respondents did not indicate their occupational category.

Fieldworkers were trained to locate selected respondents, obtain written informed consent and distribute self-administered questionnaires, which were available in English and local languages (SeSotho and Afrikaans). In cases of refusals — there were only 15 refusals — fieldworkers had a replacement list from which to select alternative respondents. Fieldworkers arranged to collect completed questionnaires and also gave the respondents a small token of appreciation. Data collection took place from January – March 2016.

### Measures

The outcome variable was measured with the statement “I am worried about contracting TB at work”. Independent variables, covering background and biographical information included questions on the respondent’s age, sex, formal education level, occupation and years of experience working as a HCW. TB knowledge was measured with ten questions (see Table 2), formulated by a clinical expert in the context of healthcare work in the Free State, South Africa. The TB Stigma scale, “Others’ External TB Stigma”, was validated [24, 25] and measures stigmatizing perceptions, attitudes and behaviours that respondents perceive as being enacted, or perceive as existing, among colleagues in relation to TB. The response options were based on a four point Likert Scale from strongly agree to strongly disagree. A four point Likert Scale, eliminates the “neutral” response option and ensure that respondents make a definite decision. Infection control was only measured, in terms of personal protective measures, by asking if there were adequate disposable respirators (N95/FFP2) in the department where the respondent worked.

### Data analysis

The data were captured, cleaned and analysed in IBM SPSS Statistics 24. Descriptive statistics were generated yielding frequency counts and percentages for categorical variables, and means and standard deviations for continuous variables. Composite scores were calculated for total TB knowledge and for the “Others’ External TB Stigma” scale. Pearson’s χ^2^ test was used to establish any association between independent variables and the outcome variable (i.e. fear of TB contagion). The independent sample t-test was used to determine if there was a difference between the means of the outcome variable (fear of TB) and continuous dependent variables (age, stigma etc.).

Multilevel modelling (i.e. a generalized linear mixed model) was used to determine if taking the clustered nature of the data into account would lead to an improvement in the model. The Akaike corrected values were compared between three models: 1) where the hierarchical nature of the data was ignored; 2) where intercepts were allowed to vary; and 3) where both intercepts and slopes were allowed to vary. Smaller Akaike corrected values indicate a better fitting model. When comparing the Akaike corrected values between models 1 and 2, the value increased when intercepts were allowed to vary (i.e. from 825.620 to 3174.821). Thus, the model that ignores the clustered nature of the data was a better fit than the model that took the clustered nature of the data into account, and allowed the intercepts to vary between the different hospitals. When comparing the Akaike corrected values between models 1 and 3, the value increased when both intercepts and slopes were allowed to vary (i.e. from 825.620 to 3220.722). Thus, the model that ignores the clustered nature of the data was again a better fit than the model that took the clustered nature of the data into account, and allowed both the intercepts and slopes to vary between the different hospitals. Therefore, taking the clustered nature of the data into account (i.e. taking into account that participants were nested within hospitals) did not result in an improvement in the fit of the model (with all independent variables added). Therefore we decided to run a binomial logistic regression and not take the clustered nature of the data into account.

Subsequently binomial logistic regression analysis was used to determine which factors were significantly associated with fear of TB infection in the workplace. All assumptions for binomial logistic regression were met. Independent variables included in the model were: sex (male/female), occupation (administration/clinical professionals/support), years as a HCW, TB knowledge, availability of disposable respirators (Yes/No), “Others’ External TB Stigma” scale, and knowing a friend, colleague and family member with TB (Yes/No). The odds ratios (ORs) together with their corresponding 95% confidence intervals (CIs) were estimated. The significance level considered for this study was 0.05.

### Ethical considerations and authorisation

Ethical clearance was obtained from the Ethics Committee of the Faculty of Health Sciences, University of the Free State (ECUFS 55/2015), and the Social Sciences Ethical Clearance Committee, University of Antwerp (SHW_15_28_04). The study was authorised by the Free State Department of Health.

## Descriptive results

### Biographic information and concerns about TB infection

The majority of respondents were female (71.7%), the average age was 43.6 years (standard deviation ±9.9), and they had been employed as a HCW for an average of 14.8 years (standard deviation ±10.7). A breakdown of education levels revealed that 42% of HCWs had a tertiary qualification and 34.7% had matric, which is South Africa’s highest school-leaving grade. Half of the respondents (50.7%) belonged to the category of clinical staff (see Table 1).

**Table 1:** Biographic characteristics and fear of contagion

	Total *n* = 882(%)	Fear TB contagion *n*(Row %)	*P* value
Sex^a^			
Male	249 (28.3)	168 (67.5)	
Female	631 (71.7)	425 (67.4)	.974
Age (Mean, SD)	43.6 (9.9)	43.2 (9.9)	.070
Education			
Secondary school and lower	206 (23.4)	117 (56.8)	
Matric	306 (34.7)	209 (68.3)	
Tertiary	370 (42.0)	267 (72.2)	.001
Occupation^a^			
Administration or management	116 (13.2)	77 (66.4)	
Clinical HCWs	446 (50.7)	317 (71.1)	
Support	318 (36.1)	199 (62.6)	.046
Years working as a HCW (Mean, SD)	14.8 (10.7)	15.5 (10.5)	.259

^a^*n* = 880

A smaller percentage of respondents (19.7%) had never been screened for TB at the hospital where they worked. The majority of respondents (67.2%) indicated that they were concerned about contracting TB at work. From the bivariate analysis we found significant associations between fear of acquiring TB at work, occupation and education. More specifically, 71.1% of clinical staff feared acquiring TB at work compared to 62.6% of support staff. Furthermore, 72.2% of HCWs with a tertiary education (i.e. a degree or diploma) feared TB contagion at work compared to 56.8% of staff who had a secondary school or lower qualification.

### TB knowledge

The respondents scored an average of 7.2 (standard deviation ±1.519) on the TB knowledge scales (minimum 0 and maximum 10) with 49.5% scoring above the average. The overwhelming majority of respondents were familiar with the four basic symptoms of TB: cough for more than 2 weeks (96.8%); unintentional weight loss (96.7%); night sweats (96.3%); and fever for more than a week (82.8%). They were less knowledgeable about TB treatment: 53.3% knew that at least four drugs were used to treat TB and 63.8% were aware that multi-drug resistant (MDR) TB could not be cured within 12 months (at the time of the fieldwork, South Africa had not implemented the 9-month treatment regimen) (see Table 2).

**Table 2:** Correct knowledge of TB diagnosis and treatment (*N* = 882)

	*n* (%)
Symptoms used to diagnose TB	
Cough for more than 2 weeks^a^	853 (96.8)
Unintentional weight loss	853 (96.7)
Fever for more than 1 week^b^	729 (82.8)
Night sweats^a^	848 (96.3)
Nausea is not used to diagnose TB^b^	464 (52.7)
TB should be treated for at least 6 months	842 (95.5)
At least 4 drugs should be used to treat TB^a^	470 (53.3)
People with TB usually become less infectious within 3 weeks of initiating appropriate treatment	640 (72.6)
People with MDR TB cannot be cured within 12 months^a^	562 (63.8)
HIV-positive HCWs can be protected from TB infection by taking IPT^*^	647 (73.4)

^a^*N* = 881

^b^*N* = 880

There was a statistically significant difference in TB knowledge scores: those HCWs who feared acquiring TB at work (mean = 7.3, standard deviation ±1.4) scored higher than those who did not fear contagion (mean = 7.0 standard deviation ±1.7) at a 95% confidence interval [− 0.215; 0.109], t(880) = − 1.977, *p* = 0.048.

### TB stigma

The respondents scored an average of 10.2 (standard deviation ±3.0) on the “Others’ External TB Stigma” scale (minimum 5 and maximum 20). More specifically, 29.5% of the respondents were in agreement that some HCWs in the hospital preferred not to eat or drink with a co-worker presumed to have TB. In addition, 25.5% of respondents agreed with the statement that some HCWs in the hospital felt uncomfortable working near to co-workers with TB (see Table 3).

**Table 3:** Others external TB stigma

	Strongly Disagree *n*(%)	Disagree *n*(%)	Agree *n*(%)	Strongly Agree *n*(%)
HCWs who are suspected of having TB are stigmatized in this hospital (*N* = 879)	216 (24.6)	499 (56.8)	130 (14.8)	34 (3.9)
Some HCWs in this hospital avoid contact with co-workers who they think may have TB (*N* = 880)	215 (24.4)	508 (57.7)	131 (14.9)	26 (3.0)
Some HCWs in this hospital do not want to eat or drink with a co-worker who they think has TB (*N* = 879)	207 (23.5)	413 (47.0)	202 (23.0)	57 (6.5)
Some HCWs in this hospital are stigmatized when others find out that they have gone for TB screening (*N* = 879)	218 (24.8)	488 (55.5)	132 (15.0)	41 (4.7)
I have noticed that some other HCWs in this hospital feel uncomfortable to work near co-workers with TB (*N* = 880)	197 (22.4)	459 (52.2)	173 (19.7)	51 (5.8)

There was a statistically significant difference in scores on the “Others’ External TB Stigma” scale: those HCWs who feared acquiring TB at work (M = 10.5, standard deviation ±3.1) scored higher than those who did not fear contagion (M = 9.5, standard deviation ±2.7) at a 95% confidence interval, [− 1.00; 0.21], t(877) = − 4.684, *p* < .001.

### Multivariate results

#### Factors associated with HCWs’ fear of acquiring TB at work

The logistic regression model was significant χ^2^(10) = 28.864, *p* < 0.005. The model explained 5.9% (Nagelkerke R^2^) of the variance in the tendency to worry about acquiring TB at work and correctly classified 69.9% of cases. After controlling for other variables in the model, three predictor variables were found to be statistically significant (*p* < .05) – occupation, availability of disposable respirators and others’ external TB stigma (see Table 4). Support staff were less likely to worry about acquiring TB compared to clinical staff (OR = 0.657, *P* = 0.041). Respondents, who indicated that there were inadequate disposable respirators at work, were 1.6 times more likely to be afraid of contracting TB at work than HCWs who indicated that there were adequate disposable respirators at work. With every unit increase on the TB stigma scale, respondents were 1.1 times more likely to fear acquiring TB at work. This implies that the more HCWs perceived colleagues to stigmatise co-workers with (presumptive) TB, the more afraid they were of acquiring TB at work.

**Table 4:** Factors associated with HCWs’ concerns about acquiring TB at work

Variables	*n* (%)	Unadjusted odds ratio (95% CI)	Adjusted odds ratio (95% CI)
Sex			
Female (ref)	425 (71.7)	1	1
Male	168 (28.3)	1.005 (0.735–1.375)	0.983 (0.668–1.448)
Occupation			
Clinical (ref)	317 (53.5)	1	1
Admin and management	77 (13.0)	0.803 (0.519–1.243)	0.936 (0.487–1.802)
Support	199 (33.5)	0.681 (0.501–0.924)	0.657 (0.439–0.982)
Years as HCW (Mean and SD)	15.5 (10.5)	0.992 (0.979–1.006)	0.989 (0.973–1.006)
TB knowledge (Mean and SD)	7.3 (1.4)	1.096 (1.000–1.201)	1.009 (0.883–1.153)
Adequate disposable respirators available			
Yes (ref)	391 (73.9)	1	1
No	138 (26.1)	1.923 (1.293–2.859)	1.571 (1.020–2.420)
“Others’ External TB Stigma” scale (Mean and SD)	10.5 (3.1)	1.123 (1.068–1.180)	1.123 (1.059–1.191)
Know of a friend with TB			
Yes (ref)	185 (32.5)	1	1
No	384 67.5)	0.934 (0.681–1.281)	0.866 (0.555–1.350)
Know of a colleague with TB			
Yes (ref)	127 (22.6)	1	1
No	435 (77.4)	1.055 (0.745–1.495)	1.262 (0.806–1.975)
Know of a family member with TB			
Yes (ref)	143 (25.0)	1	1
No	428 (75.0)	0.939 (0.669–1.317)	0.943 (0.602–1.477)

## Discussion

In short, we found that the majority of HCWs feared occupationally-acquired TB and that being a clinical HCW, not having adequate disposable respirators available and perceiving co-workers to stigmatise colleagues with (presumptive) TB were all significantly associated with the fear of occupationally-acquired TB. More specifically, most (67.2%) of the 882 HCW respondents were afraid of contracting TB at work, with management, administration and support staff less likely to fear acquiring TB at work than clinical professionals. This is to be expected, as clinical professionals such as doctors, nurses and allied staff work in close contact with patients. The finding that HCWs are afraid of acquiring TB at work is supported by earlier research [7, 17–19]. These fears are not unfounded: evidence of the nosocomial transmission of (DR)TB to HCWs [2, 15, 16, 26] suggests that they are at least twice as likely to contract TB [13]. There are also strong indications that TB infection control measures are poorly implemented in healthcare facilities [7–9, 11, 12, 27, 28]. All this points to HCWs not being safe in their working environment. Perception of risk as well as actual risk are closely tied to the fear of contagion [29]. In clinical environments with high levels of TB bacilli, a certain level of fear of contagion is understandable, arguably even necessary. However, elevated levels of fear of contagion, and factors that generate this fear, have negative effects that seriously detract from creating and maintaining a health-enabling working environment.

Although at the lowest level of TB infection control, the lack of personal protective equipment (i.e. disposable masks) was also one of the drivers of fear of contagion, a fear that can be reduced if infection control measures at all levels are adequately implemented. Research has found that adequate infection control measures may decrease the annual TB incidence among HCWs by as much as 49, 27, and 81% in countries with low, intermediate, and high TB incidence, respectively [2]. Evidently, there are supply gaps, but the question remains: would HCWs actually use disposable respirators if they were readily available? Other research has reported infrequent use of disposable respirators even among those working in high risk areas [7–9, 11, 12]. This not only calls for improvements in supply of but also need for sensitisation about personal protection against TB at the hospitals, while keeping in mind that this is the lowest level of infection control and that priority should be given to the higher levels that focus on, amongst others, separating and triaging of coughing patients and adequate ventilation.

Previous research highlighted that fear of acquiring TB was a predictor of TB stigma [30]. Our study found that perceiving co-workers to stigmatise colleagues with (presumptive) TB was positively associated with fear of occupationally-acquired TB. De Andrade et al. [31] maintain that this fear is rooted in historical and social memories of the disease, marked by stigma, segregation and exclusion. For example, in the Victorian era TB was somewhat romanticized in film and fiction—the disease was labelled in a less clinical way as “consumption”, and ‘victims’ ‘faded away’. A slow shift in perceptions of TB saw patients become labelled as TB “suspects” or “defaulters”— terms with strong criminal undertones [1]. In recent years there has been a move back to more neutral terminology such as “presumptive” TB patients rather than TB “suspects”. Nevertheless, HCWs continue to fear TB, and as noted by von Delft et al. [20] when diagnosed with TB, they often seek treatment in secret.

The strengths of our study lie in the large-scale quantitative design, random selection of participants and especially developed scales to measure different types of TB stigma. However, as with all research, ours has limitations. The pre-intervention data were cross-sectional in nature, which means that at this point we can only infer an association and not causation. We did not ask participants if they had TB and our infection control question only addressed the lowest level in the hierarchy of infection control, namely the use of personal protective measures. As with most self-reported measures, some level of response bias is likely. Given the sensitive nature of stigma, respondents may have chosen to answer the stigma-related questions in a socially desirable manner. We attempted to deal with this by: using unique identifying numbers and not respondent names on the questionnaires; keeping the signed informed consent letters separate from the questionnaires; and assuring respondents that their answers would be anonymised and reported as aggregated data. Finally, it was beyond the scope of our study to explore in-depth reasons why HCWs fear acquiring TB at work: qualitative enquiry and explanatory models are two possibilities for further work in this regard.

## Conclusions

Being a professional clinical HCW, not having adequate disposable respirators available and seeing/perceiving co-workers stigmatise colleagues with (presumptive) TB were all significantly associated with the fear of occupationally-acquired TB. Campaigns to destigmatise TB, as well as appropriate TB infection control education and measures, are necessary to alleviate HCWs fears of acquiring the disease in the workplace. Ultimately, all levels of the healthcare system will have to be engaged to address HCWs’ risk, as well as their fear, of TB contagion — from the individual level of HCWs themselves, through community levels such as local hospitals and their patients, to the systemic level where, inter alia, implementation plans are formulated and their effectiveness monitored.

## Data Availability

Data from the current study are available from the corresponding author on reasonable request and once clearance is obtained from the University of the Free State, University of Antwerp and Free State Department of Health.

## Abbreviations

CI: Confidence intervals
DRTB: Drug-resistant tuberculosis
HCW: Healthcare worker
LTBI: Latent TB infection
MDRTB: Multi-drug resistant tuberculosis
OR: Odds ratios
TB: Tuberculosis
